# Fracture-Related Infection of a Distal Femur Open Fracture Treated With a Supracutaneous Locking Plate

**DOI:** 10.7759/cureus.65909

**Published:** 2024-07-31

**Authors:** Chieng Zhin Liang, Ahmad Faiz Mohamed Khalil, Nik Kamarul Arif, Syed Syafiq, Mohd Hisam Muhamad Ariffin

**Affiliations:** 1 Orthopaedics and Traumatology, Universiti Kebangsaan Malaysia Medical Centre, Kuala Lumpur, MYS

**Keywords:** multidisciplinary care, open fracture, distal femur fracture, supracutaneous locking plate, fracture-related infection

## Abstract

Fracture-related infection (FRI) is a challenging complication in open fractures. It can cause major disability to patients and a burden to the public health sector. A multidisciplinary approach is required to eradicate infection and improve the quality of life for patients. We present a case of an FRI in an open fracture of the distal femur treated using a supracutaneous locking plate, which is an uncommon technique. This technique yields excellent outcomes in controlling local infection and providing satisfactory stability, especially for a peri-articular distal femur fracture with FRI. Therefore, supracutaneous plating using a locking plate can be considered an alternative option to conventional external fixations in managing FRIs.

## Introduction

Infection is one of the major complications in the treatment of open fractures [1]. Infections after internal fixations range from 1.6% in closed fractures to 8% in open fractures [2]. This may cause major disability to patients and affect their quality of life. Furthermore, it also increases the socioeconomic burden on the public health sector.

Diagnosis for fracture-related infection (FRI) was standardized in 2018 based on recent updates provided by the FRI consensus group, which included confirmatory and suggestive criteria [3]. This update provided guidelines for standard treatment pathways and outcome measures [4]. FRI requires multidisciplinary teamwork and collaboration to improve patient outcomes.

A supracutaneous plating technique using a locking compression plate (LCP) as an external fixator was described in metaphyseal and diaphyseal compound fractures of the tibia [5,6]. This technique provides relative stability which allows secondary fracture healing. Studies have shown that supracutaneous plating techniques in compound fractures of the tibia produced good clinical outcomes and low complication rates [6]. This technique is patient-friendly as the plate is low profile and light. It also can prevent joint stiffness as it does not cross the joint.

In this study, we report a case of an FRI in an open fracture of the distal femur managed using a supracutaneous locking plate.

## Case presentation

**Figure 1:**
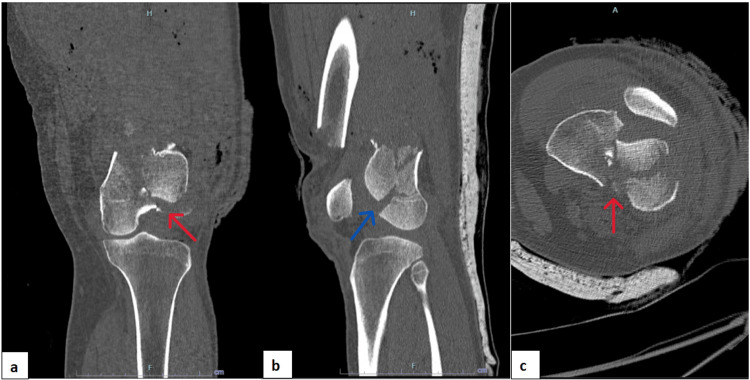
Computed tomography scan of the left knee showing a comminuted fracture of the left distal femur with an intercondylar split and Hoffa fragment at the lateral femoral condyle. (a) Coronal view. (b) Sagittal view. (c) Axial view. The red arrows show intercondylar split, and the blue arrow shows the Hoffa fragment.

A 41-year-old male with underlying diabetes mellitus was involved in a motor vehicle accident. While riding his motorcycle, he skidded and fell into a drain. Post-trauma, he sustained a Grade 3A open fracture of the left distal femur. The patient was initially admitted to a private hospital. Imaging of the left knee showed a comminuted fracture of the left distal femur with an intercondylar split and a Hoffa fragment over the lateral femoral condyle (Figure 1). The patient was treated with triple antibiotics including intravenous (IV) cefuroxime, IV metronidazole, and IV gentamicin. Wound debridement and arthrotomy washout of the left knee were performed on the same day as the accident.

Second-look wound debridement, cross-knee external fixation, and screw fixation to address the intercondylar split and Hoffa fragment were performed a few days after the first operation. Second-look wound debridement is ideally performed 48-72 hours after the first operation, especially in high-energy injury to ensure all devitalized and unhealthy tissue is adequately removed. Subsequently, the wound healed and the patient was discharged with oral antibiotics after two weeks of admission.

**Figure 2:**
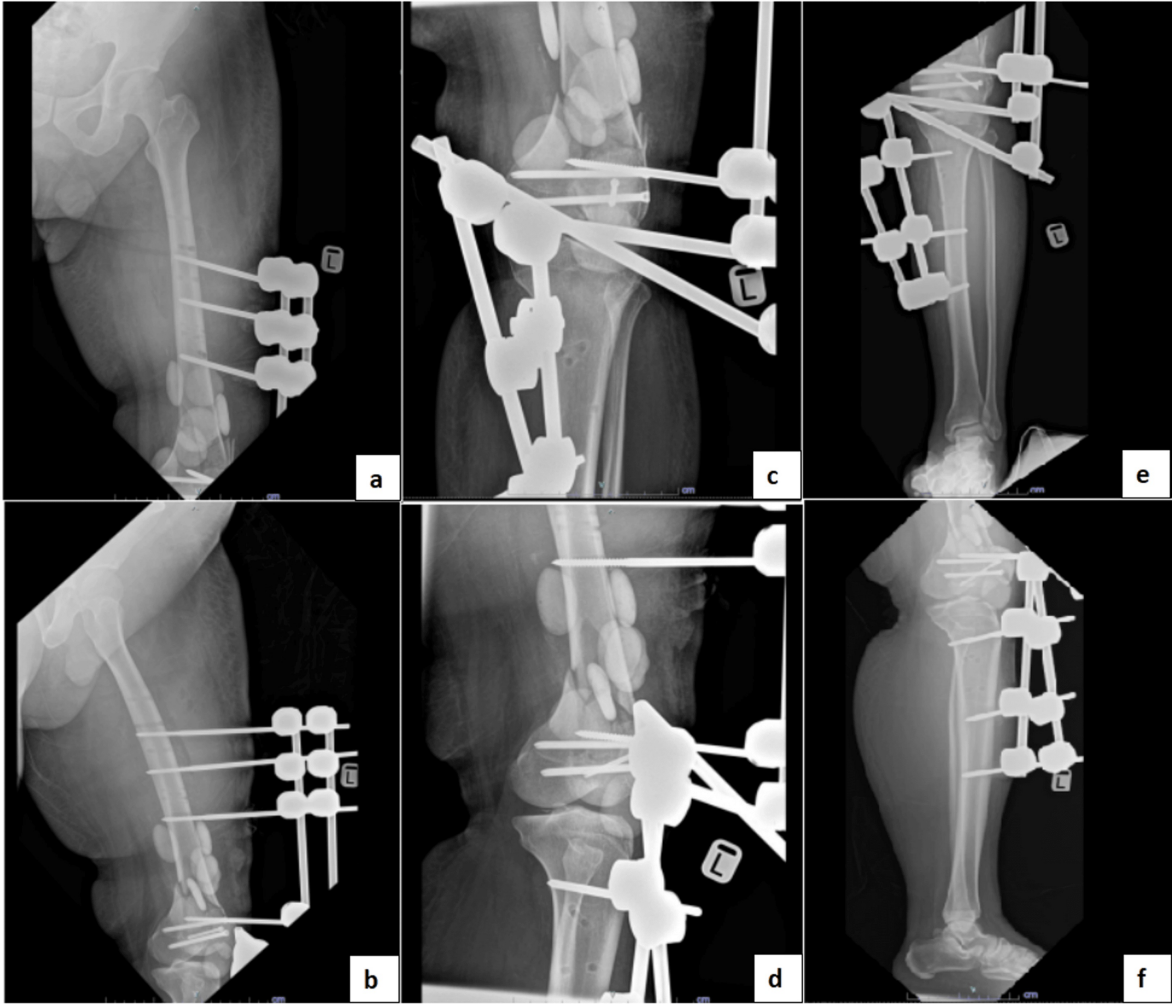
Radiographs after the readjustment of left knee cross-knee external fixator and gentamicin bead insertion. (a) Femur anteroposterior view. (b) Femur lateral view. (c) Knee anteroposterior view. (d) Knee lateral view. (e) Tibia/fibula anteroposterior view. (f) Tibia/fibula lateral view.

However, one week after the second-look wound debridement, the patient developed a pin-site infection over the proximal pins of the external fixator. He was re-admitted and wound debridement, left knee arthrotomy washout, gentamicin bead insertion, and relocation of external fixator pins were performed (Figure 2). Intraoperative cultures yielded extended-spectrum beta-lactamase *Klebsiella pneumonia* growth, sensitive to antibiotics such as meropenem. Subsequently, the patient was transferred to our center for the continuation of care.

**Figure 3:**
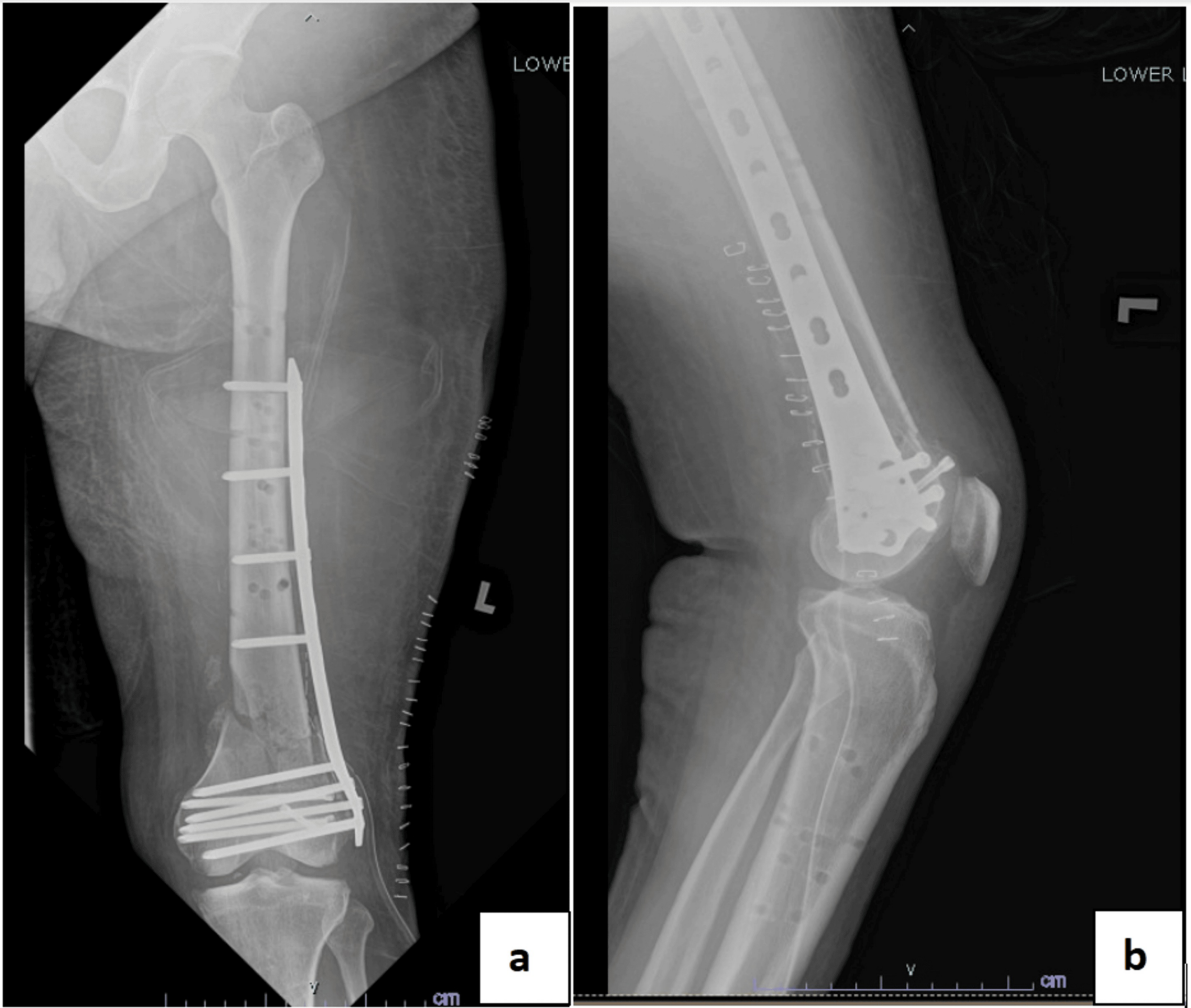
Radiograph of the left femur showing the left distal femur locking plate, performed after the removal of the left cross-knee external fixator. (a) Anteroposterior view. (b) Lateral view.

An infectious disease physician was consulted for antibiotic treatment. The patient was treated with IV meropenem for a total of three weeks, followed by IV cefuroxime for another three weeks. The wound healed and septic parameters improved. One month after the last wound debridement, the cross-knee external fixator was removed and the pin sites were cleaned and debrided. The patient was put on an above-knee backslab for a week before a titanium distal femur locking plate was inserted (Figure 3).

Unfortunately, the wound breakdown occurred one week after the plating of the left distal femur. Subsequently, the patient underwent wound debridement and collatamp insertion. Collatamp is a gentamicin-impregnated collagen matrix that is used to control local infection. Intraoperative tissue and bone cultures produced mixed organisms. The patient was treated as FRI of the left distal femur. However, we decided to keep the implant given it was newly inserted and early debridement could remove immature biofilm. He resumed IV meropenem for six weeks after consultation with the infectious disease physician.

**Figure 4:**
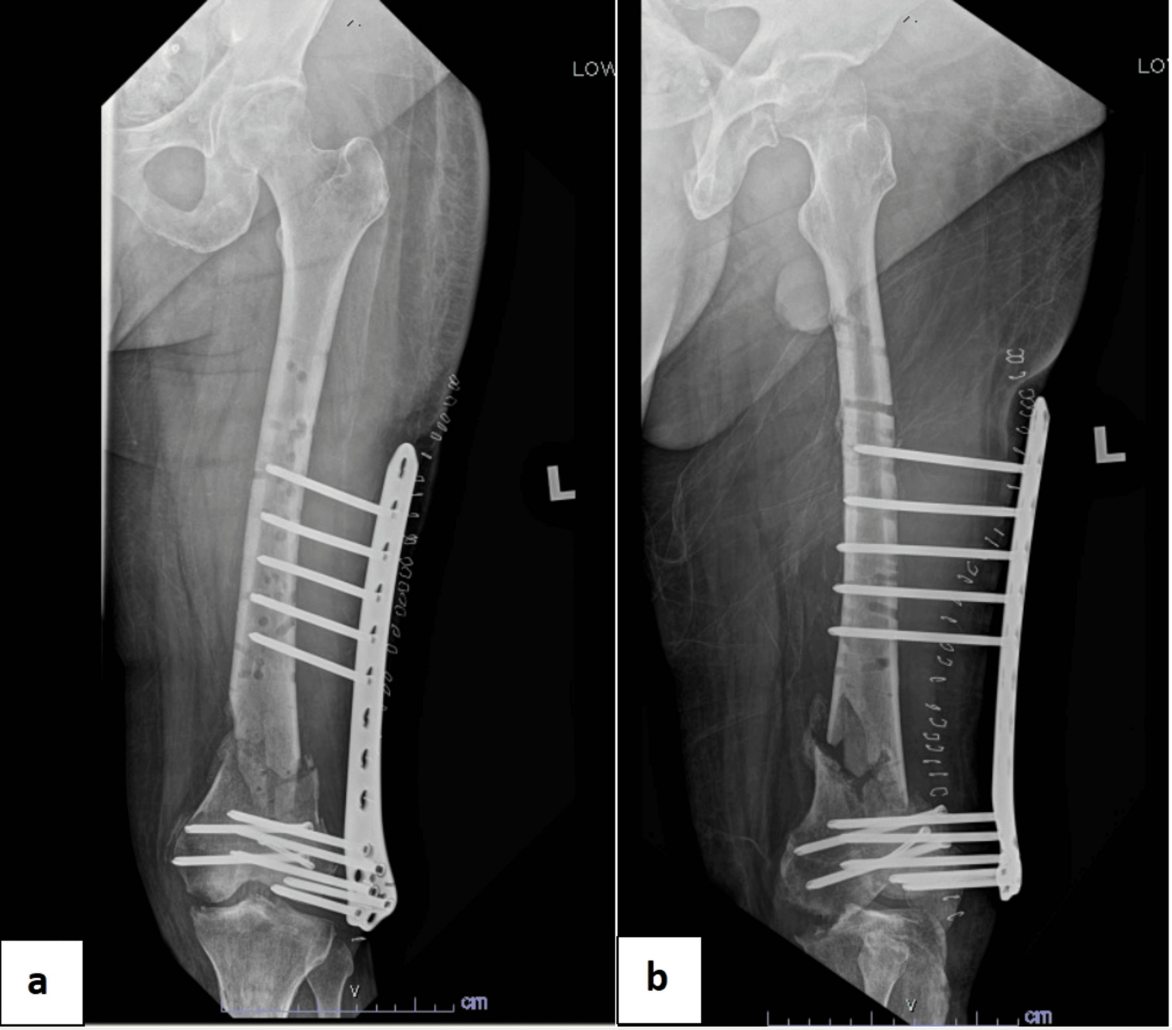
Radiograph of the left femur after supracutaneous plating of the left distal femur using a distal femur locking plate. (a) Anteroposterior view. (b) Lateral view.

Upon completion of the IV meropenem course, sinus formation occurred with persistent pus discharge over the distal part of the wound at the lateral aspect of the thigh. A CT scan over the left femur showed a left distal femur fracture with concerning evidence of osteomyelitic changes. Thorough wound debridement and removal of the left distal femur locking plate were performed. Debridement included sinus tract excision, removal of the infected tissue, and large-volume wound irrigation. Intraoperative findings showed the presence of a viscous, shiny film which could represent biofilm formation with no implant loosening. The fracture was stabilized with a supracutaneous distal femur locking plating which was positioned over the anterolateral aspect of the left thigh (Figure 4). Intraoperative tissue cultures and bone cultures produced methicillin-resistant *Staphylococcus aureus* (MRSA), which could be caused by prolonged antibiotic exposure, an immunocompromised state due to underlying diabetes mellitus, or contamination from skin commensals. The patient was treated with IV vancomycin for two weeks.

**Figure 5:**
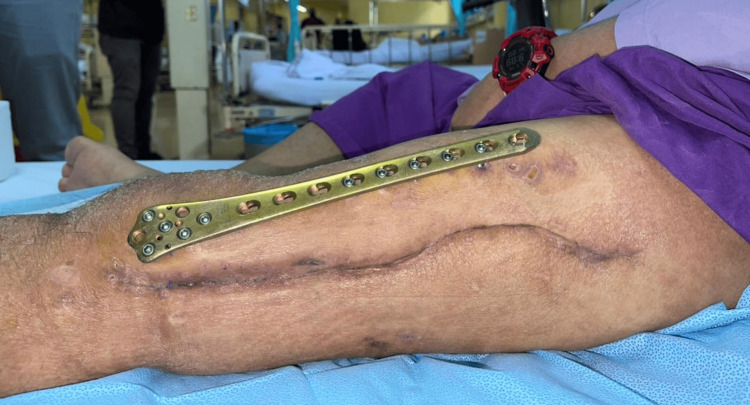
Clinical pictures of the left thigh showing the healed wound over the lateral aspect of the thigh before discharge.

Subsequently, septic parameters improved and the wound healed (Figure 5). The patient was discharged with oral antibiotics tablet rifampicin and tablet bactrim, to be taken for 10 weeks, as suggested by the infectious disease physician. Upon discharge, the patient was able to ambulate with a walking frame, and the range of motion of the left knee was 0-70 degrees.

**Figure 6:**
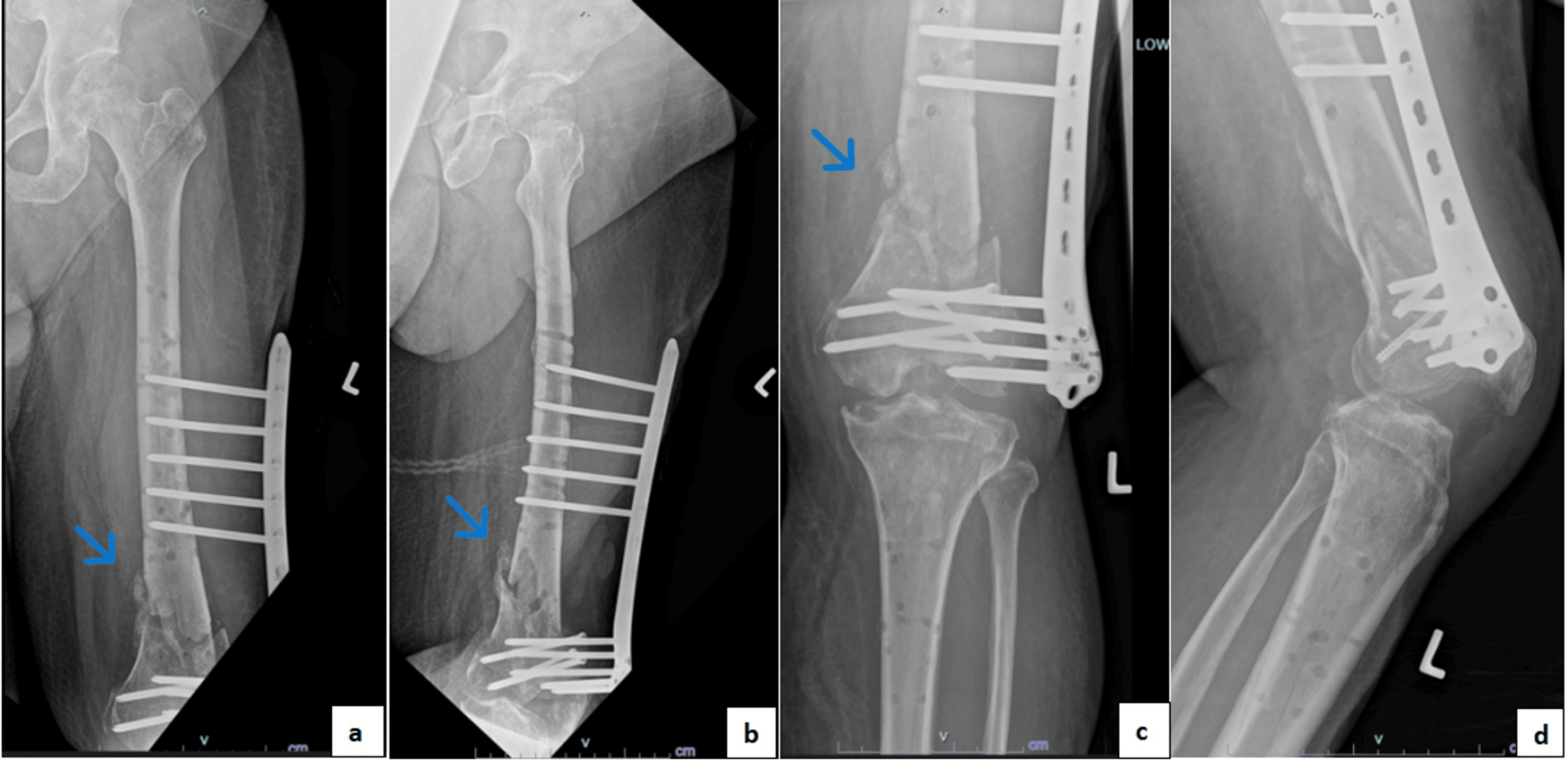
Radiographs at three months post-supracutaneous plating showing callus formation at the fracture site. (a) Femur anteroposterior view. (b) Femur lateral view. (c) Knee anteroposterior view. (d) Knee lateral view. The blue arrows show callus formation.

**Figure 7:**
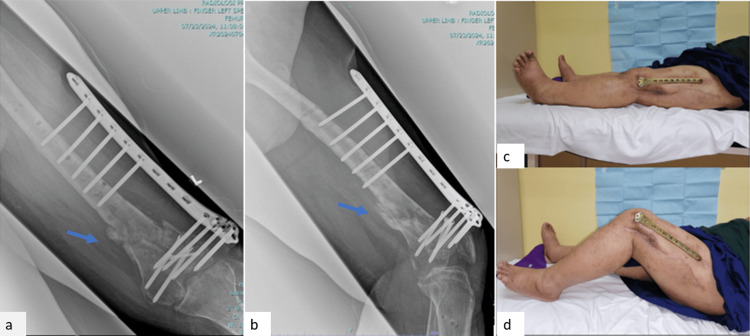
Radiographs and clinical photos at the sixth-month follow-up showing abundant callus formation and a good knee range of motion. (a) Femur radiograph anteroposterior view. (b) Femur radiograph lateral view. (c) Knee in full extension. (d) Knee in full flexion. The blue arrows show callus formation at the fracture site.

The three-month post-supracutaneous plating follow-up radiographs (Figure 6) showed callus formation at the distal femur fracture site. Clinically, the surgical wound healed well, with no signs of local infection. The patient was allowed to partially weight-bear with the supracutaneous locking plate. Six-month follow-up radiographs showed abundant callus formation, and, clinically, the patient has a good knee range of motion (Figure 7). There was no limb length discrepancy for bilateral lower limbs.

## Discussion

FRI is a challenging complication in cases of open fracture [3,4]. The risk of infection following an open fracture is determined by several factors such as the initial severity of the injury, the extent of wound contamination, and patient factors [7]. This patient sustained a Grade 3A Gustilo-Anderson open fracture over his left distal femur. Grade 3A carries a 4% risk of wound infection, while Grade 3B and Grade 3C carry infection risks of 42% and 52%, respectively [8]. A more recent publication showed a broader infection rate in Grade 3 fractures (2.8-40.5%). Despite initial wound debridement being performed within 24 hours of injury and adequate antibiotic administration, this patient developed an FRI as early as two weeks after trauma. Another contributory factor for FRI in this patient could be due to his immunocompromised state caused by underlying poorly controlled diabetes mellitus. Studies have shown that diabetes mellitus has a higher rate of post-traumatic infection compared to those without diabetes [9].

Diagnostic criteria for FRIs were published in 2018 by the FRI consensus group [3]. The introduction of diagnostic criteria provides standard guidelines for orthopedic surgeons to manage FRI patients. It consists of confirmatory and suggestive criteria [4]. This patient fulfilled the confirmatory criteria, as there was the presence of sinus and purulent discharge from the wound. Additionally, intraoperative cultures produced high-virulence organisms such as *Klebsiella pneumonia* and MRSA. Furthermore, serum inflammatory markers such as C-reactive protein (CRP) and total white count (TWC) were also increased at the initial presentation. CRP was 10.9 mg/dL and TWC was 20.6 × 10^9^/L.

FRIs require a multidisciplinary approach to improve patient clinical outcomes [4]. For this patient, an infectious disease physician was consulted regarding the choice and duration of antibiotics. The patient required multiple courses of intravenous antibiotics such as IV meropenem and IV vancomycin for the eradication of infection. A physiotherapist was also involved since the beginning of the treatment to provide postoperative rehabilitation to improve the patient’s functional outcomes. Upon discharge, the patient was able to ambulate using a walking frame. In addition, a dietitian, diabetes educator, and pharmacist also played important roles throughout the management of the FRI.

The internal fixator was removed given a persistent purulent discharge from the wound, despite multiple wound debridements and courses of antibiotics. A CT scan showed a distal femur fracture with evidence suggestive of osteomyelitis at the femoral condyle. The fracture was stabilized using a distal femur locking plate via a supracutaneous plating technique after implant removal. However, the screws which addressed the intercondylar split and Hoffa fragment were left undisturbed to prevent displacement of fracture at the articular surface. The distal femur locking plate was positioned at the anterolateral aspect of the left distal thigh, rather than directly lateral, for a few reasons. First, there is limited availability of locking screw length to achieve adequate cortices purchase for fracture stabilization at the distal femur. This is because the patient’s muscle bulk at the lateral aspect of the thigh was larger than the anterolateral aspect of the thigh due to swelling. Second, it is to facilitate dressing, as there was a wound directly over the lateral aspect of the distal thigh from a previous surgery. Third, the anterolateral position of the plate can prevent irritation of the iliotibial band, which is located laterally over the thigh. This can allow the patient to start early range of motion exercises on his left knee.

An LCP used as an external fixator was first described by Kerkhoffs et al. for the treatment of open fractures, inferior non-union, and arthritis [5]. Supracutaneous plating using locking plates is gaining popularity because of a few advantages. It is less bulky which can accommodate the patient to wear trousers [5,6,10]. In addition, it is also lighter and of low profile [6,11]. This allows the patient to ambulate and travel with less inconvenience. The LCP also provides greater versatility for screw insertion to obtain the greatest bone purchase at the distal fragment [5]. Furthermore, it can prevent knee joint stiffness, compared to a spanning knee external fixator, by allowing early range of motion exercises of the knee. In addition, supracutaneous plates also allow easier assessment of callus formation with radiographs when compared to other implants [6,10,12].

The challenges we faced for this patient included difficulty in dressing the pin site as the plate was located very close to the skin. Without proper regular pin-site dressing, the patient might be predisposed to pin-site infection and pin loosening. Moreover, we also had intraoperative difficulty due to the limited availability of screw lengths for the distal fragment. Therefore, we could only purchase near-cortex for distal fragment screws. Despite this, this fixation is considered stable as the locking plate itself could provide angular stability, and multiple screws were inserted at the distal fragment.

## Conclusions

FRI is one of the common and major complications in open fractures. It is challenging to simultaneously eradicate infection and maintain the stability of the fracture. FRIs require multidisciplinary approaches to achieve good clinical outcomes. In addition, FRIs can also cause stress and psychosocial impacts on patients, especially when patients need to undergo multiple surgeries and prolonged hospital stays. Therefore, appropriate surgical technique is crucial to determine the outcome of patients diagnosed with FRI. In this case, we shared our experience of using LCPs in supracutaneous plating to treat distal femur FRI.

LCPs can be used as external fixators for comminuted distal femur fractures with FRI. It provides satisfactory stability, allows wound healing, and re-use of the internal fixator to external fixation. Moreover, it is less cumbersome when compared to conventional external fixators, and was accepted by the patient. Furthermore, it also facilitates postoperative rehabilitation by allowing early knee movements, which can improve the patient’s functional outcomes. Further follow-up is required for this patient to monitor for fracture union and true assessment of infection control or eradication.
